# Author Correction: Checkpoint blockade immunotherapy reshapes the high-dimensional phenotypic heterogeneity of murine intratumoural neoantigen-specific CD8^+^ T cells

**DOI:** 10.1038/s41467-018-05468-y

**Published:** 2018-07-26

**Authors:** M. Fehlings, Y. Simoni, H. L. Penny, E. Becht, C. Y. Loh, M. M. Gubin, J. P. Ward, S. C. Wong, R. D. Schreiber, E. W. Newell

**Affiliations:** 1 Agency for Science, Technology and Research (A*STAR), Singapore Immunology Network (SIgN), 8 A Biomedical Grove, Singapore, 138648 Singapore; 2 Department of Pathology and Immunology, School of Medicine, Washington University in St. Louis, 660 South Euclid Avenue, St. Louis, MO 63110 USA

Correction to: *Nature Communications*; 10.1038/s41467-017-00627-z; published online: 15 Sep 2017

The original version of this Article omitted a declaration from the competing interests statement, which should have included the following: ‘R.D.S. is a cofounder, stock holder, and scientific advisory board member of Jounce Therapeutics and Neon Therapeutics, and a member of the scientific advisory boards of BioLegend, Constellation, Lytix, and NGM. He also received research funding from Janssen and Agios.’. This has now been corrected in both the PDF and HTML versions of the Article.

