# Supplementary material for: Checkpoint blockade immunotherapy reshapes the high-dimensional phenotypic heterogeneity of murine intratumoural neoantigen-specific CD8+ T cells
Source: Nat Commun. 2017 Sep 15;8:562. doi: 10.1038/s41467-017-00627-z (PMC5601925; doi:10.1038/s41467-017-00627-z)
Supplement: Supplementary file 1 — Supplementary Information [file 41467_2017_627_MOESM1_ESM.pdf]

Description of Supplementary Files

File name: Supplementary Information
Description: Supplementary figures and supplementary tables

File name: Peer Review File

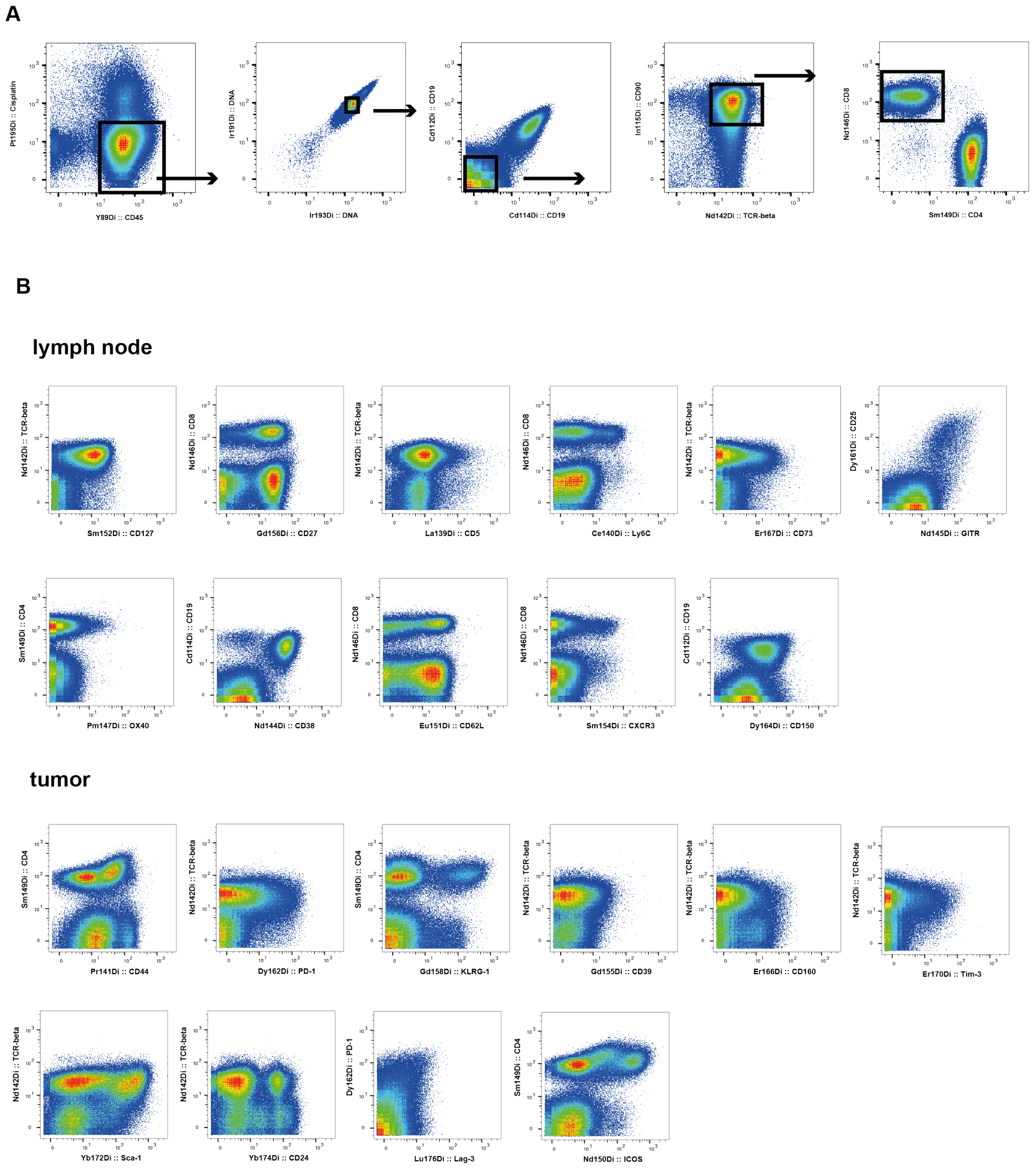

Supplementary Figure 1. Antibody staining assessed by mass cytometry. (A) General gating strategy for the identification of CD8^{+} T cells by mass cytometry**.** Shown are biaxial dot plots from the draining lymph nodes of tumour bearing mice. **(B)** Quality of surface marker stainings assessed by mass cytometry. Shown are biaxial dot plots from the draining lymph nodes or tumours from tumour bearing mice.

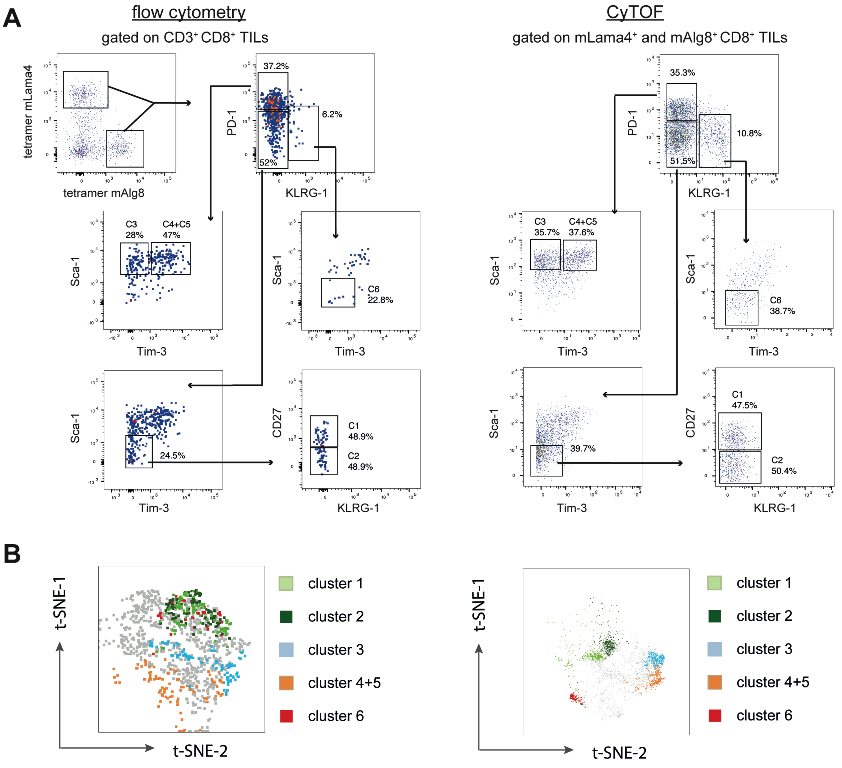

Supplementary Figure 2. Flow cytometry analysis of neoantigen-specific TILs. **(A)** Manual gating on neoantigen-specific CD8$^+$ T cells from individual tumours of untreated tumour bearing mice analyzed by flow cytometry in comparison to data obtained by mass cytometry. Sequential biaxial dot plot gating strategy according to the signature markers characteristic for clusters C1-C6 segregated by t-SNE identified in Fig. 2A. Data shown is one representative of at least three independent experiments. **(B)** Visualized t-SNE maps displaying the distribution of tetramer positive antigen-specific T-cell clusters assessed by flow and mass cytometry.

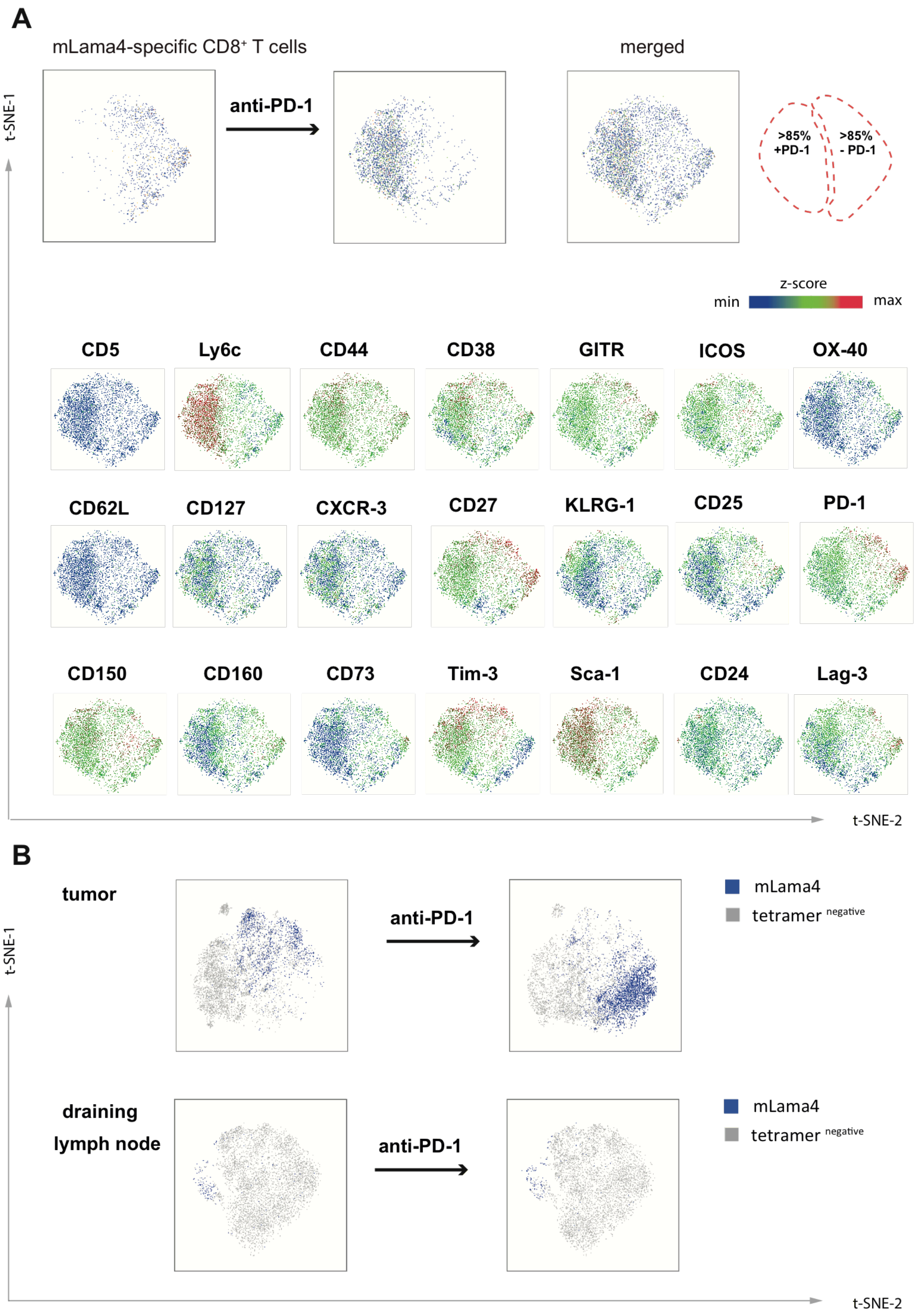

Supplementary Figure 3. PD-1 blocking affects phenotypes of neoantigen-specific TILs. (A) Anti-PD-1 treatment shifts mLama4-specific TILS from different clusters towards a novel position on a two-dimensional t-SNE plot. Cluster specific phenotypes are represented by normalized expression intensities of relevant marker molecules displayed on the merged t-SNE map. The vast majority of cells derived

from anti-PD-1 or isotype treated tumour bearing mice occupy the left or right moiety of the t-SNE plot respectively. **(B)** Visualized t-SNE map highlighting the distribution of mLama4-specific T cells and tetramer negative cells within tumours and draining lymph nodes. In contrast to tumours, T-cell clusters specific for mLama4 from draining lymph nodes can be identified within similar positions on a two-dimensional t-SNE plot, irrespectively of anti-PD1 treatment. Data shown is one representative of three independent experiments (n=5 mice per group).

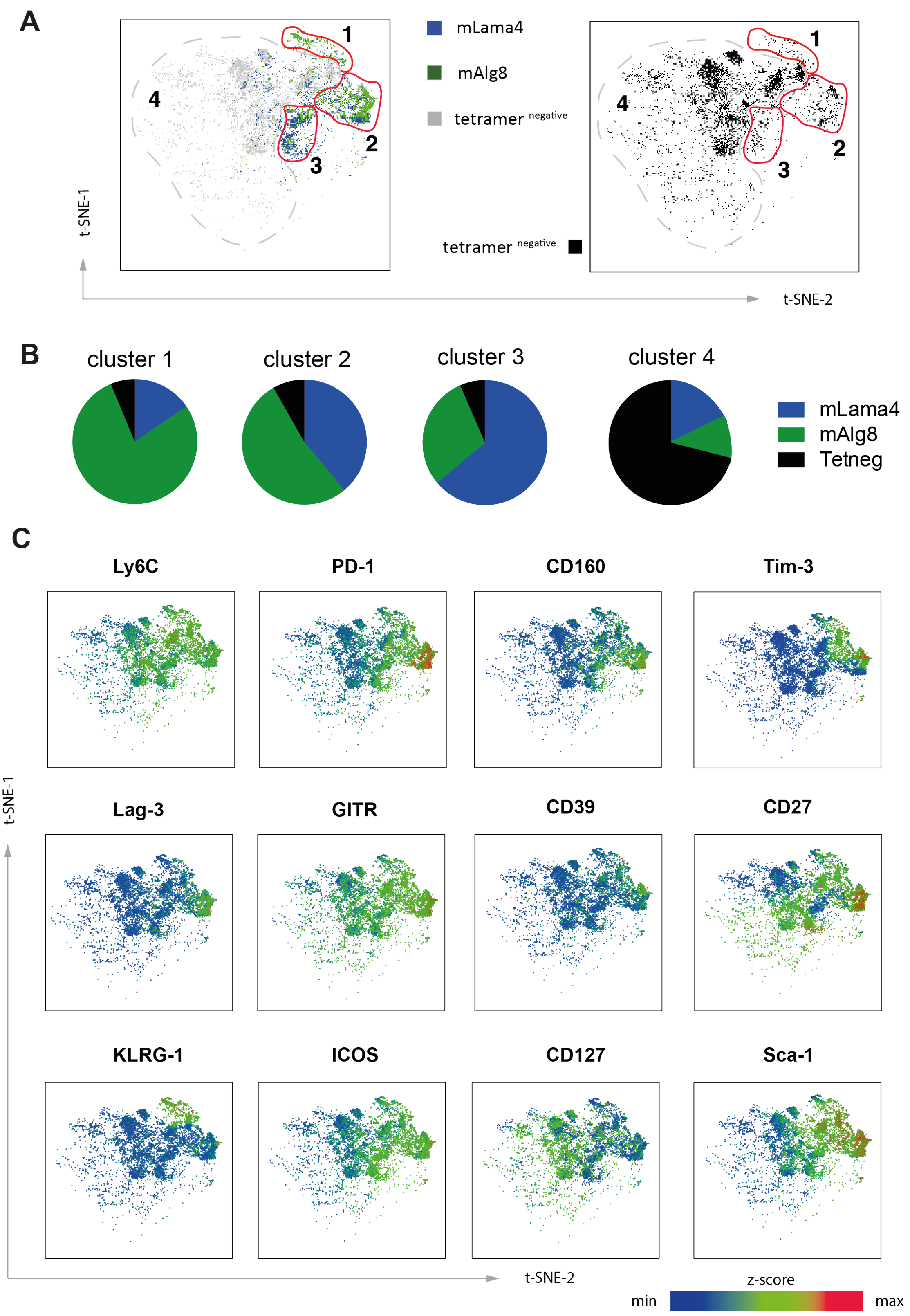

Supplementary Figure 4. Phenotypic overlaps between tumour-specific and tetramer negative TILs. (A) Manual gating identified four overlapping clusters

segregated by t-SNE that consist of tumour derived neoantigen-specific $CD8^+$ T cells (mLama4 and mAlg8) and tetramer negative $CD8^+$ T cells. **(B)** Pie charts displaying percentages of antigen-specific and tetramer negative T cells for each cluster identified in (A). **(C)** Normalized expression intensities of relevant marker molecules displayed on the t-SNE map.

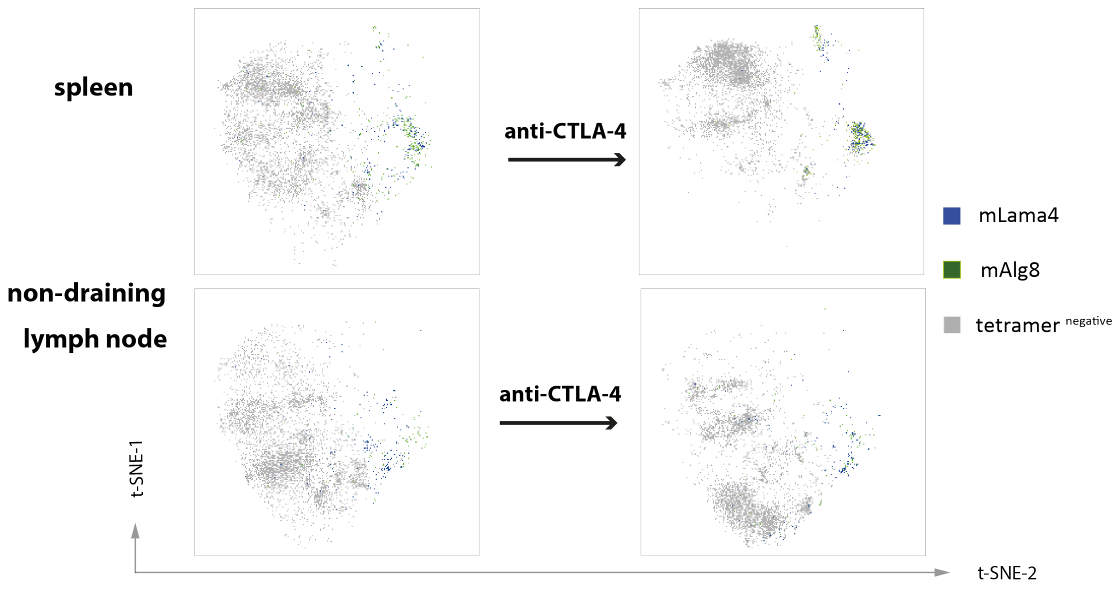

Supplementary Figure 5. Distribution of neoantigen-specific and tetramer negative TILs. CD8^{+} T cells from spleens and non-draining lymph nodes in anti-CTLA-4 and control (isotype) mAbs treated mice (related to Fig. 4A, n=5 mice per group).

Supplementary Table 1: Epitope candidates

peptide	sequence	peptide	sequence
Gpr108	SVRSYSRL	EG668618	IVPFYPAL
Slco1c1	ILYLFAST	Col9a1	VAFSYKGM
Dsc1	TSLKYKIL	Olfr1225	VSWAGAFL
Olfr1239	ISDMYPLL	Spop	LSPVFSAM
Lama4	VGFNFRTL	Nrxn3	SMYKYRNR
Nf1	SITAYLNL	Tpm2	TTLLFSFL
Olfr1121	SYYRYVAI	Sspo	SSLSYCSV
3425401B19Rik	VNSTYSPL	Prss34	VIENFMPL
Ak7	ATYVVASV	4932438H23Rik	ISFLDMAL
Acsf3	VGATYVML	Sart3	MWLEYSNL
Vmn1r90	VITLFEGL	Tnfsf14	VSLSLVLL
Batf	VVYSAHAL	Calcoco1	QLYQFPYV
Ccny	YTIKFVAL	Zfp619	RGFSYLRI
Sema6d	PMYRYYRL	ENSMUSG00000078985	SNTAYMQL
Sbf2	FNYLYSPL	Atp13a5	IGYVYAQK
Serpinb9f	SSVEFIFL	Vkorc1	TAISFSRV
Oprd1	ITALYSAL	Lass4	LNYAHFRL
Cpn1	SAFLHLLL	Ube2C	LSLEFPSV
6430548M08Rik	KVYLYTRL	Olfr683	LSYSFILM
Fpr2	TTILYLNL	Olfr684	LSLLYIVL
Gpr98	VNFCYVLV	Olfr12	MAYDRFMAM
Apob	SSNVYSNL	Olfr1030	VQFYFFIAM
EG667741	ANWPYTNL	Cntnap5b	VRFVRFVPL
EG624584	SLFPFFNV	Olfr73	VQFFFFSTF
Olfr53	TSVLYSFV	Cpn1	SAFLHLLLL
Olfr849	VSVLFFRV	Ncapg	IHYLKYSMV
LOC328483	STTVYPTL	Olfr1204	PIYYFLAYL
Abcd2	YSPKFGSL	Adam29	YNYFWSYTL
Olfr1440	VIFVVASV	Cx3cr1	ISIDRYIAI
Alg8	ITYTWTRL	Trpv6	VMLERKLPL
BC018465	ISFSLAQM	Gpr98	ANFTFSAWL
Olfr1204	IYYFLAYL	Olfr168	VVTFYYAHF
Olfr988	MIYVVTLL	Olfr845	VSLFYVTGL
Olfr1424	IYFLLRNL	Lrrn1	LSYIHSLAF
Olfr168	VTFYYAHF	Psg23	IVYSNRSLL
Olfr169	AHFVYTYL	Fahd2a	TFHTFCPL
Neurl1B	VIYTCGPM	Lrrn2	SHRAFAGL
Mlc1	VIPNFQIL	ENSMUSG00000054758	VPLSYNRL
Fbxl4	STGCFARL	Lrrc8a	SFVIFYGL
ENSMUSG000000 78806	FSPGFRRL	Gm98	YYFHFYSL
4930404A10Rik	KAQQFANL		

Supplementary Table 2: Antibodies used for mass cytometry staining panel

antibody	clone	provider
CD90	T24/31	BioXcell
CD45	30-F11	Fluidigm
CD19 Q-dot	6D5	Life technologies
CD5	53-7.3	Biolegend
Ly6C	HK1.4	Biolegend
CD38	90	Biolegend
CD44	IM7	BioXcell
TCR-beta	H57-597	Biolegend
GITR	DTA-1	Biolegend
CD8a	53-6.7	Biolegend
OX-40	OX-86	Biolegend
CD4	H129.15	Biolegend
ICOS	C358.4A	Biolegend
CD62L	MEL-14	Biolegend
CD127	A7R34	Biolegend
CXCR-3	CXCR3-173	Biolegend
CD39	5F2	Biolegend
CD27	LG.7F9	eBioscience
KLRG-1	2F1	Biolegend
CD25	PC61	Biolegend
PD-1	29F.1A12	Biolegend
CD150	TC15-12F12.2	Biolegend
CD160	7H1	Biolegend
Tim-3	B8.2C12	Biolegend
CD24	M1/69	Biolegend
Sca-1	D7	Biolegend
Lag-3	C9B7W	Biolegend
CD73	TY/23	BD
